# Draft Genome Sequence of a Multidrug-Resistant Strain of *Enterococcus faecalis*, PM01, Isolated from the Nest of an American Bushtit, *Psaltriparius minimus*

**DOI:** 10.1128/genomeA.00017-17

**Published:** 2017-03-16

**Authors:** Saika Esani, John V. H. Constable, Tricia A. Van Laar

**Affiliations:** Department of Biology, College of Science and Mathematics, California State University, Fresno, Fresno, California, USA

## Abstract

Pathogenic microorganisms associated with avian nests may detrimentally impact parental health and nest success for the nest primary users, potentially neighboring avian or terrestrial species, including humans. Here, we report the genome sequence of *Enterococcus faecalis* strain PM01, isolated from a failed nest of American bushtits, *Psaltriparius minimus*.

## GENOME ANNOUNCEMENT

The microbiome of the nest is interesting, as it likely contributes to the horizontal transfer of the parent microbiome to offspring through incubation and feather linings that may influence nest success. However, nests may also house pathogenic microbes, and several studies have shown that bird feces may be a significant source of disease for humans (1–3), but there are also concerns surrounding the role of feces, and the general nest microbiome, as a pathogen source that reduces avian reproductive success (4). Beyond the direct role of the nest microbiome in egg viability (5), nestling growth (6), effects on secondary avian or mammalian nest residents (7), and implications for neighboring avian and terrestrial species (1, 8), nests may also serve as natural reservoirs for both benign and pathogenic microbes.

*Enterococcus faecalis* is a Gram-positive bacterium commonly associated with the gastrointestinal tract. *E. faecalis* is a relatively common cause of infection and is associated with high levels of antimicrobial resistance (9) and virulence factor production, including cytotoxins (10).

*E. faecalis* strain PM01 was isolated from the nest of an American bushtit, *Psaltriparius minimus*, on the campus of California State University, Fresno. The nest contained the remains of three nestlings covered in feces, indicating poor parental care and nest failure. The nest area containing the offspring remains and feces was sampled using a sterile swab premoistened in storage solution (10 mM Tris, 1 mM EDTA, 0.05% [vol/vol] Tween 20 [pH 8.0]). The sample was swabbed on the surface of a tryptic soy agar plate supplemented with 5% sheep’s blood for isolation of colonies. Colony PM01 was grown overnight in Luria-Bertani broth. Total genomic DNA was purified using the Wizard Genomic DNA purification kit (Promega, Madison, WI, USA), and the strain was identified as *E. faecalis* through 16S rRNA sequencing (11). In addition to isolate identification, we determined its antimicrobial resistance profile, as this bacterium was found on a college campus and potentially came into contact with humans. Using a 96-well-plate assay (12), we identified that *E. faecalis* PM01 was resistant to a number of antibiotics, including ampicillin, chloramphenicol, meropenem, methicillin, and spectinomycin at concentrations greater than 200 μg/ml.

*De novo* genome sequencing of *E. faecalis* PM01 was provided by MR DNA (Shallowater, TX) using an Illumina MiSeq with 250-bp paired-end reads. The sequence was processed and assembled using the A5-miseq assembly pipeline (13). Assembly yielded a genome with 2,832,817 bp and 37.3% G+C content. There were 29 contigs with an *N*_50_ contig size of 295,750 bp. The genome was annotated using the RAST annotation server (14) and was predicted to contain 2,618 protein-coding sequences and 46 noncoding RNAs.

Further study of the nest microbiome will provide important insights into the role microbes play in the ecology of avian reproduction.

### Accession number(s).

This whole-genome shotgun project has been deposited at DDBJ/ENA/GenBank under the accession no. MSHQ00000000. The version described in this paper is version MSHQ01000000.
